# Mechanisims of asthma and allergic disease – 1086. Bacteria-derived extracellular vesicles as an important causative agent for asthma and COPD

**DOI:** 10.1186/1939-4551-6-S1-P82

**Published:** 2013-04-23

**Authors:** Young-Koo Jee, You-Sun Kim, Eun-Jeong Choi, Won-Hee Lee, Seng-Jin Choi, Tae-Young Roh, Jae-Sung Park, Zhou Zhu, Young-Yull Koh, Yong Song Gho, Yoon-Keun Kim

**Affiliations:** 1 Internal Medicine, Dankook University College of Medicine, Cheonan, South Korea; 2 Life Science and Division of Molecular and Life Sciences, Pohang University of Science and Technology (POSTECH), Pohang, South Korea; 3 Asthma and Allergy Center, Johns Hopkins University School of Medicine, Baltimore, USA; 4 Pediatrics, Seoul National University College of Medicine, Seoul, South Korea

## Background

Many bacterial components in indoor dust can evoke inflammatory pulmonary diseases. Bacteria secrete nanometer-sized vesicles into the extracellular milieu, but it remains to be determined whether bacteria-derived extracellular vesicles in indoor dust are pathophysiologically related to inflammatory pulmonary diseases. We evaluated whether extracellular vesicles (EV) in indoor air are causally related to the pathogenesis of asthma and/or emphysema.

## Methods

EV were prepared by sequential ultrafiltration and ultracentrifugation from indoor dust collected from a bed. Innate and adaptive immune responses were evaluated after once or 4 weeks airway exposure of EV, respectively.

## Results

Vesicles 50-200 nm in diameter were present (102.5microgram [based on protein concentration]/g dust) in indoor dust, and inhalation of 1 microgram of these vesicles for 4 weeks caused neutrophilic pulmonary inflammation. Additionally, polymyxin B (an antagonist of endotoxin, a cell wall component of Gram-negative bacteria) reversed the inflammation induced by the dust EV. Indoor dust harbors *Esherichia coli*-derived vesicles; airway exposure to the vesicles for 4 weeks induced neutrophilic inflammation and emphysema, which were partially eliminated by the absence of IFN-gamma or IL-17. Interestingly, serum dust EV-reactive IgG1 levels were significantly higher in atopic children with asthma than in atopic healthy children and those with rhinitis or dermatitis. Moreover, serum dust EV-reactive IgG1 levels were also elevated in adult asthma or COPD patients than in healthy controls.

## Conclusions

EV in indoor dust, especially derived from Gram-negative bacteria, appear to be an important causative agent in the pathogenesis of asthma and/or emphysema.

